# A translational EEG-based approach to assess modulation of long-lasting NMDAR-dependent synaptic plasticity

**DOI:** 10.1007/s00213-019-05341-w

**Published:** 2019-08-07

**Authors:** Jeffrey S. Burgdorf, E. P. Christian, L. Sørensen, P. K. Stanton, K. Leaderbrand, T. M. Madsen, M. A. Khan, R. A. Kroes, J. R. Moskal

**Affiliations:** 1 Aptinyx Inc., 1801 Maple Ave, Suite 4300, Evanston, IL 60201 USA; 2 Falk Center for Molecular Therapeutics, Department of Biomedical Engineering, Northwestern University, Evanston, IL 60201 USA; 3 Cell Biology & Anatomy, New York Medical College, Valhalla, NY USA

**Keywords:** LTP, Mismatch negativity, EEG, Auditory-evoked potentials, NMDA receptors

## Abstract

**Background:**

NYX-2925 is a novel *N*-methyl-d-aspartate receptor (NMDAR) modulator that has been shown to facilitate both NMDAR-dependent long-term potentiation (LTP) in vitro and learning and memory in vivo.

**Objective:**

The present studies examine the effects of NYX-2925 on NMDAR-dependent auditory LTP (aLTP) in vivo.

**Methods:**

NMDAR-dependent aLTP and NMDAR-dependent auditory mismatch negativity (MMN) was measured, as well as changes in resting-state qEEG power.

**Results:**

NYX-2925 (1, 10 mg/kg PO) increased aLTP 1 h after auditory tetanus measured by the post- minus pre-tetanus difference waveform 140–180 ms post tone onset. NYX-2925 (0.1, 1 mg/kg PO) facilitated MMN measured by the difference waveform (i.e., deviant minus standard tones). NYX-2925 (0.1, 1, 10 mg/kg PO) also enhanced resting-state alpha qEEG power. Conversely, the NMDAR glutamate site antagonist CPP (10 mg/kg IP) reduces alpha power and MMN and produces an opposite effect as NYX-2925 on aLTP.

**Conclusions:**

Together, these data suggest that the activation of the NMDAR by NYX-2925 enhances synaptic plasticity in vivo*,* which may both reduce symptoms of neurological disorders and serve as a biomarker for drug effects. This is the first demonstration of a long-lasting (1-h post-tetanus) effect of NMDAR modulation on synaptic plasticity processes in vivo using a noninvasive technique in freely behaving animals.

## Introduction

NMDA receptor (NMDAR) activity is critical for synaptic plasticity. For example, NMDARs are required for the induction of some forms of long-term potentiation (LTP), a form of a long-lasting increase in synaptic strength that is a putative substrate for learning and memory (Luscher and Malenka 2012). The induction, but not long-term maintenance, of LTP at many excitatory synapses in hippocampal and medial prefrontal cortex (MPFC) slices is blocked by the NMDA glutamate receptor antagonist APV, and by NMDAR channel blockers such as MK-801 and ketamine (Bliss and Collingridge 2013; Davis et al. 1992). In addition, NMDAR activation promotes LTP (Zhang et al. 2008). Once LTP has been established, in a process which includes the insertion of AMPA receptors into synapses in a protein synthesis-dependent manner, the increased synaptic effect that is the expression of LTP is not blocked by NMDAR antagonists (Luscher and Malenka 2012).

In vivo, learning acquisition and long-term memory formation of hippocampus- and MPFC-dependent tasks are also blocked by NMDAR antagonists (Burgdorf et al. 2011; Davis et al. 1992). In vivo electrical stimulus-induced LTP is also NMDAR-dependent, can be blocked by the NMDAR antagonist APV (Morris 1989), and is facilitated by application of the NMDAR agonist d-cycloserine (DCS; (Hopkins et al. 2013)). NMDAR activation is essential for a large portion of LTP induced by tetanization with sound or visual stimuli, as recorded by sensory-evoked potentials via scalp EEG electrodes (Sanders et al. 2018). NMDAR activation with DCS facilitates short-term potentiation (Forsyth et al. 2015), and inactivation with CPP (an analog of APV) inhibits the induction of LTP (Clapp et al. 2006). Here, in order to determine whether NMDAR modulation can facilitate LTP in vivo, a modulator of the NMDAR with long-lasting properties was used.

NYX-2925 ((2S,3R)-3-hydroxy-2-((R)-5-isobutyryl-1-oxo-2,5-diazaspiro[3.4]octan-2-yl)butanamide) is a novel NMDAR modulator (Khan et al. 2018; Moskal et al. 2017). Supported by robust preclinical data in animal models of neuropathic pain (Ghoreishi-Haack et al. 2018), NYX-2925 is currently being investigated in two phase II clinical studies for the treatment of painful diabetic peripheral neuropathy and fibromyalgia. Previous studies demonstrated that NYX-2925 is a member of a novel class of NMDAR-specific modulators that facilitate NMDAR-dependent processes associated with learning and memory (Khan et al. 2018). Ex vivo, NYX-2925 enhances hippocampal and MPFC LTP and inhibits the induction of long-term depression (LTD) at concentrations that facilitate NMDAR current (Khan et al. 2018). Persistent changes in long-lasting plasticity are also seen 24 h post dosing in vivo*,* as measured by facilitation of ex vivo hippocampal LTP and mature dendritic spine morphology that far outlasts drug presence (Khan et al. 2018). In vivo, NYX-2925 enhances NMDAR-dependent positive emotional learning and this effect is blocked by the NMDAR glutamate site antagonist CPP. Oral doses of NYX-2925 in vivo that increase learning and memory produce CSF concentrations that enhance NMDAR current in vitro (Khan et al. 2018).

Therefore, an in vivo translational EEG approach was used to evaluate the long-lasting effects of NYX-2925-induced NMDAR modulation on synaptic plasticity changes. This approach should be directly applicable to similar studies conducted in human subjects and provide new insights into NMDAR-mediated synaptic plasticity mechanisms and their role in CNS disorders (Clapp et al. 2012; Clapp et al. 2005), such as centrally mediated chronic pain (e.g., fibromyalgia), post-traumatic stress disorder, and cognitive impairment.

The experiments in this study were designed to test whether NYX-2925 could enhance auditory-evoked responses 1 h after auditory tetanus (aLTP). As part of this experiment, NMDAR-dependent mismatch negativity (MMN), the auditory event-related potential to a deviant tone compared with a standard tone, was also examined, as well as resting quantitative EEG (qEEG) power across the alpha frequency band.

## Materials and methods

### Animals

Male 2–3-month-old Sprague-Dawley (SD) rats from Envigo (USA) were used. Rats were housed in Lucite cages with aspen wood chip bedding, maintained on a 12:12 light:dark cycle (lights on at 6 am) and given ad libitum access to Teklad lab chow (Envigo, USA) and tap water throughout the study. All experiments were approved by the Northwestern University Animal Care and Use Committee.

### Surgeries, data acquisition, and analysis

Rats were anesthetized with isoflurane (5% induction and 2–3% maintenance; 15–20 min total duration) and implanted with skull screws to record cortical EEG (Pinnacle, USA). Animals were given 7 days to recover before the start of testing. Auditory event-related potentials (ERPs) were recorded from a frontal cortex skull screw using a cerebellar skull screw as a ground/reference. EEG signals were captured via a tethered system (Pinnacle, USA). Data were acquired at 1 kHz using an A&M (USA) amplifier with a high (0.1 Hz)- and low-pass (100 Hz) filters and digitized using Data Wave (USA) acquisition software. Data were analyzed using Brain Products Analyzer 2 software (Germany).

Animals were dosed with NYX-2925 (0.1, 1, 10 mg/kg PO; Sai Life Sciences, India) or 0.5% carboxymethylcellulose (Sigma, USA) in distilled deionized water (Millipore, USA). NYX-2925 was administered orally so that these preclinical data can be more directly compared with a parallel human study with NYX-2925 that also used oral dosing. An additional group of rats received the NMDAR glutamate site antagonist CPP (10 mg/kg IP; Sigma USA) or distilled deionized water vehicle. This dose of CPP was shown to block the learning and memory effects of NYX-2925 (Khan et al. 2018) as well as block in vivo hippocampal LTP (Abraham and Mason 1988).

### Quantitative EEG acquisition

Within 5 min of dosing, rats were tethered and placed into individual sound-attenuated chambers under dim illumination. For the first 60-min post-dosing, no external stimulation was given (resting state). qEEG data from the first 60-min post-dosing was analyzed across the entire period using a Hanning window (2 s, 0.5-Hz resolution). Relative (1.5 to 40 Hz) voltage spectral density (μV/Hz) was used for the analysis.

### MMN paradigm

**Fig. 1:**
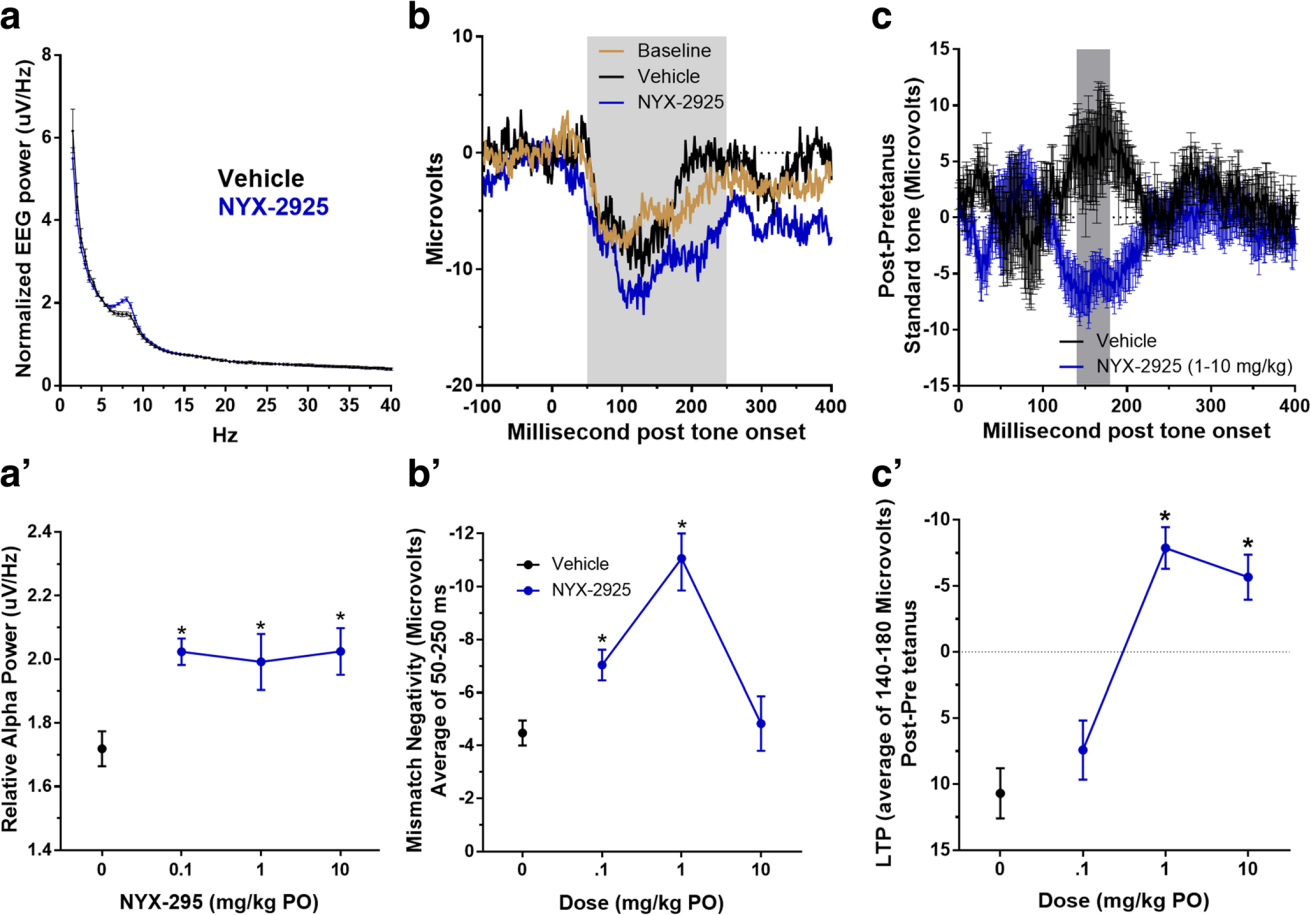
NYX-2925 enhances resting alpha power (qEEG), mismatch negativity (MMN), and auditory-induced long-term potentiation (aLTP). (A, A’) Average power spectral density plots showing that NYX-2925 (0.1, 1, 10 mg/kg PO) enhanced alpha power (7–8.5 Hz) compared with vehicle without affecting power in the other EEG bands (data not shown). (B, B’) Grand average MMN waveforms for showing that NYX-2925 (0.1, 1 mg/kg PO) enhanced MMN compared with vehicle from 50 to 250 ms after tone onset. (C, C’) Average post-pre tetanus waveforms show that NYX-2925 (1, 10 mg/kg PO) enhanced aLTP 140–180 ms after tone onset. This range was used given that this was the region in which the vehicle group showed the largest post-pre tetanus change. (B) LTP was enhanced in the 1 and 10 mg dose groups (*p* < .05). Using a similar methodology (Clapp et al. 2006), an NMDAR antagonist has been shown to inhibit sensory-induced LTP. Mean ± SEM. *p < .05 Bonferroni post hoc test vs. vehicle following a one-way ANOVA. *n* = 7–10 per group

Rats were placed in cylindrical test cages (30.5 cm diameter, 30.5 cm high clear Acrylic cylinder; Pinnacle USA). Testing consisted of standard (6 kHz) or deviant (8 kHz) tone pips (50-ms duration) and an interstimulus interval of 350 ms, presented in a pseudorandom manner, with at least 2 standards occurring in a row. Tones were presented at 85 dB using a speaker (Avisoft, Germany) placed on the floor of the cage. One trial consisted of 1800 standard tones and 200 deviant tones (total, 2000 tones). Averaged standard and deviant waveforms were baselined to the pre-stimulus period (− 50 to 0 ms before tone onset). A difference wave was obtained by subtracting the standard from the deviant waveform and was used as the measure of MMN. The range used for calculating MMN was determined based on the pre-dose MMN difference grand average waveform for all of the animals tested (50–250 ms) as shown in Fig. 1B. MMN was calculated as the average microvolt value from 50 to 250 ms after the tone onset from the deviant-standard difference wave.

### aLTP induction stimulus paradigm

aLTP was induced by a high-frequency train (3 × 10 Hz for 5 min; 3000 tones) of auditory stimuli (6 kHz, 50-ms duration, 85 dB), using a paradigm similar to the one described by Clapp and colleagues (Clapp et al. 2006). MMN testing occurred immediately before tetanus (pre-tetanus) and 1 h after tetanus (post-tetanus). Post- minus pre-tetanus difference waves were generated to determine the range (in milliseconds) in which aLTP occurred for both the standard and deviant tone as defined by the largest mean difference and statistical difference (within-subject *t* test) in the vehicle group pre- vs. post-tetanus. Using this criterion, aLTP occurred within the range of 140–180 ms after the tone onset in the vehicle group based on the average of the standard and deviant tone waveforms, which is consistent with the range (ms) and generalization (both standard and deviant tone) seen in humans with aLTP using a MMN paradigm (Kompus and Westerhausen 2018). The average microvolt value across 140–180 ms for both the standard and deviant tone was used to calculate effects of aLTP on both the NYX-2925 and vehicle groups and determine possible drug effects.

### Experimental timeline

**Fig. 2:**
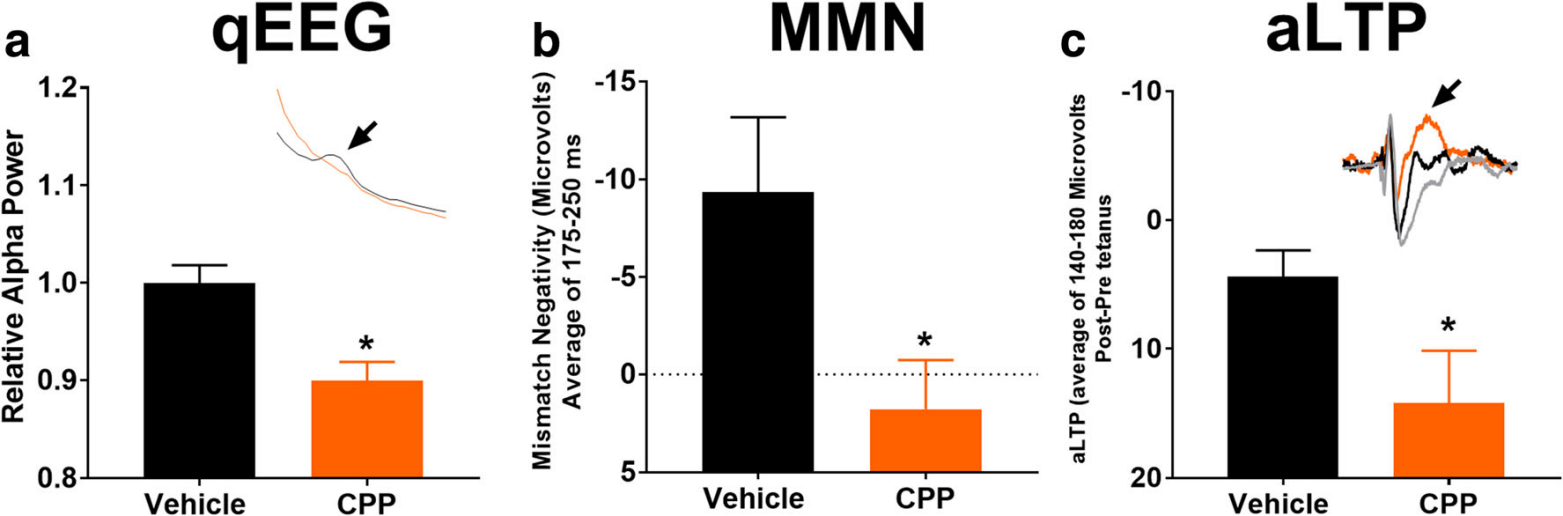
The NMDAR glutamate site antagonist CPP inhibited resting alpha power, MMN, and aLTP. CPP inhibited resting alpha power (A), MMN (B), and increased aLTP-induced positivity (C). NYX-2925 (Fig. 1) had the opposite effect on all three of these measures. Insets: (A) alpha peak (arrow), showing the CPP nearly eliminates the alpha peak; (C) effect of tetanus on aLTP from the grand average of the standard and deviant waveforms with gray being the pre-tetanus waveform. CPP produces a near maximal positive deflection across the aLTP window (140–180 ms; arrow). *p < .05 one-way ANOVA, CPP vs. vehicle. *n* = 9–10 per group

On the day before testing, animals received a habituation session of qEEG and MMN. These data were used to randomize animals into treatment groups. On the testing day, 10 min of qEEG was first collected followed by MMN. Animals were then dosed with NYX-2925 (0, 0.1, 1, 10 mg/kg PO) or the NMDAR glutamate site antagonist CPP (0, 10 mg/kg IP). Immediately after dosing, 1 h of qEEG was collected and used for the qEEG measures shown in Figs. 1A, A’ and 2A. Immediately after qEEG testing, rats received MMN testing, and these data are shown in Figs. 1B, B’ and 2B. Animals then received 3 auditory tetani (each consisting of a 5-min tetanus with a 10-min inter-tetani interval). One hour after the first tetanus, the MMN paradigm was administered again and post-pre tetanus auditory-evoked responses were used for calculating aLTP (data shown in Figs. 1C, C’ and 2C).

### Statistical analysis

The effect of NMDAR activation on qEEG and MMN variables was analyzed by a separate one-way analysis of variance (ANOVA) test with a null hypothesis of no difference in the dependent variable, with respect to the independent variable of each of the 4 doses (NYX-2925 0.1, 1, and 10 mg/kg PO, and vehicle). For the aLTP study, a within-subject component was also used (post-pre tetanus difference scores were calculated). All statistical tests were two-sided with significance set at *p* < .05. For any ANOVA test indicating significance, pairwise comparisons using *t* tests were applied between doses. These pairwise comparisons were adjusted for multiplicity using the Bonferroni correction.

Likewise, CPPs were analyzed against vehicle using the one-way ANOVA. All analyses were conducted with Statview (USA) software. Individual datapoints from unstable EEG recordings were excluded from the analysis.

## Results

qEEG measures revealed a significant modulation of the alpha range (7–8.5 Hz) (*F*(3,31) = 5.72 *p* < .01) in response to acute exposure with NYX-2925. All NYX-2925 dose levels showed a significantly increased mean alpha voltage density compared with vehicle, confirmed by the Bonferroni adjusted multiple comparisons (NYX-2925 0.1, 1, 10 mg/kg PO vs. vehicle *p* < .05), shown in Fig. 1A’.

Grand average voltage spectral density plots for NYX-2925 (0.1, 1, 10 mg/kg) and vehicle are shown in Fig. 1A.

Average MMN amplitude (50–250 ms) was significantly modulated between groups (*F*(3,25) = 11.59 *p* < .001) in response to acute exposure of NYX-2925. NYX-2925 (1 mg/kg PO) showed a significantly enhanced MMN amplitude compared with vehicle, confirmed by the Bonferroni adjusted multiple comparisons (NYX-2925 1 mg/kg PO vs. vehicle *p* < .001), shown in Fig. 1B’.

Average waveforms for the deviant-standard difference wave (MMN) for NYX-2925 (0.1, 1 mg/kg) and vehicle are shown in Fig. 1B.

The aLTP protocol, inducing auditory synaptic plasticity, modulated the standard tone waveform, measured by the mean amplitude from 140 to 180 ms (*F*(3,32) = 24.62 *p* < .001). NYX-2925 (1, 10 mg/kg PO) significantly increased the mean negativity in this range compared with vehicle, confirmed by the Bonferroni adjusted multiple comparisons (NYX-2925 1, 10 mg/kg PO vs. vehicle *p* < .001), shown in Fig. 1C’.

Average post- minus pre-aLTP waveforms for the active doses (0.1 and 1 mg/kg) and vehicle are shown in Fig. 1C.

The NMDAR glutamate site antagonist, CPP, produced the opposite effects on each of the EEG variables (Fig. 2) used to evaluate positive modulation of the NMDAR with NYX-2925. CPP inhibited resting alpha power from 7 to 8.5 Hz (*F*(1,18) = 14.05 *p* < .01; Fig. 2A), whereas NYX-2925 enhanced alpha power (Fig. 1A). CPP also inhibited the latter segment of the MMN waveform (175–250 ms post tone onset) (*F*(1,18) = 5.89 *p* < .05; Fig. 2B), which NYX-2925 preferentially enhanced (Fig. 1B). CPP also increased aLTP-induced positivity (140–180 ms post tone onset) (*F*(1,16) = 4.68, *p* < .05; Fig. 2C), whereas NYX-2925 produced negativity across this same range (Fig. 1C).

EEG response during the auditory tetanus stimulation was also examined. The EEG was visualized by transforming data and recorded during auditory tetanus, to the time-frequency domain using the Morlet wavelets (Morlet parameter = 3, optimized for the best time sensitivity). This inspection showed that a 6–7-Hz band best followed the auditory stimulation in that a power in the band was seen in a burst that occurred approximately 10 per second and were evenly spaced out, resembling the 10-Hz tone presentation (50-ms tone duration). These 6–7-Hz bursts occurred primarily within the first 160 s of the 5-min tetanus. Thus, a Fourier analysis of the first 160 s of the tetanus was employed and relative power was measured from 6 to 7 Hz. The 6–7-Hz band was significantly modulated between groups (*F*(2,54) = 19.3, *p* < .05), with NYX-2925 (0.1, 1, 10 mg/kg PO) showing a significant enhancement (*p* < .05) and CPP showing a significant decrease (*p* < .05) compared with vehicle, suggested by the Bonferroni adjusted multiple comparisons.

## Discussion

This is the first demonstration that positive modulation of the NMDAR facilitates NMDAR-dependent sensory-evoked LTP in vivo. Across auditory and visual modalities, NMDAR-dependent LTP has typically been defined as persisting for at least 1 h after sensory tetanus. Potentiation of this duration requires NMDAR activation, is blocked by NMDAR antagonists, is AMPAR-dependent, and requires protein synthesis, whereas short-term potentiation does not typically require NMDAR activation and involves only transient activation of AMPAR, not insertion of new AMPARs into the postsynaptic membrane (Malenka and Bear 2004). Given that NYX-2925 enhances NMDAR activity, which leads to long-lasting synaptic plasticity, learning acquisition, and long-term memory formation (Khan et al. 2018), it is an ideal molecule for testing in an in vivo NMDAR-dependent LTP assay.

The ability of NYX-2925 to enhance NMDAR-dependent plasticity in vivo was independently confirmed by the MMN paradigm and qEEG studies in response to the aLTP protocol. NMDAR-dependent MMN was facilitated by NYX-2925 both acutely and 1 h after aLTP. Using this paradigm, NMDAR antagonists have been shown to reduce (Ehrlichman et al. 2008; Sivarao et al. 2014), and glycine (which, among other actions, is a co-agonist at the NMDAR) to facilitate, MMN (Greenwood et al. 2018). The present studies with NYX-2925 further validate the NMDAR dependence of MMN.

The alpha band has been defined as the primary resting-state EEG rhythm, and using this definition, alpha power is positively correlated with increased attention (Klimesch 1999). In addition, enhancing alpha power with repetitive transcranial magnetic stimulation (Klimesch et al. 2003; Romei et al. 2010) or neurofeedback training (Hanslmayr et al. 2005; Zoefel et al. 2011) can improve cognitive task performance. Thus, resting alpha synchronization has predictive power for subsequent sensory experience or cognitive processing, whereas attention to sensory stimuli desynchronizes evoked alpha rhythm and this desynchronization is greater with increasing attention to stimuli (Engel et al. 2001; Silvanto et al. 2008). NYX-2925 enhanced resting alpha power (7–8.5 Hz). As alpha power may reflect an attentional vigilance state that primes an animal for sensory processing (e.g., MMN and aLTP), increased resting/spontaneous alpha power in the rat EEG may represent a mechanism by which NYX-2925 enhances learning and memory through facilitating attention.

Resting alpha power, MMN, and aLTP are NMDAR-dependent phenomena given that they are inhibited by the NMDAR glutamate site antagonist CPP. The NMDAR-dependent variables showed a dynamic activation, seen as the inverted U-shaped dose-response relationship (Fig. 1B’, C’), suggesting that various brain circuits and mechanisms are affected at different dose levels of NYX-2925 exposure. They may be modulated by different NMDAR receptor subtypes given that NYX-2925 shows a different dose-response across each of these assays, and NYX-2925 enhances NMDAR subtype activity with different potencies (NR2B > NR2A > NR2C = NR2D) as reported in Khan et al. (2018). Therefore, it is possible that alpha power and MMN may be more NR2B-mediated than aLTP. Interestingly, the late temporal range for MMN (175–250 ms post tone onset) that is most sensitive to habituation across testing days (Fig. 1B) is also the most sensitive to NMDAR activation (Figs. 1B and 2B). The NMDAR dependence of aLTP also is seen in response to the tetanus, with positive NMDAR modulation facilitating the entrainment of the EEG response to stimulus and NMDAR antagonisms inhibiting this response. Lastly, MMN and aLTP occur across a different time window with MMN being later (175–250 ms) than aLTP (140–180 ms), suggesting that these processes may underlie different neuronal populations.

NYX-2925 is a member of a novel platform of NMDAR modulators, four of which are now in human clinical studies. This is the first study demonstrating that NYX-2925 acts as an NMDAR modulator in vivo and establishes a method for measuring the long-lasting effects of NMDAR activation using a method readily translatable to noninvasive studies in humans. Such studies will provide new insights into NMDAR-mediated synaptic plasticity mechanisms and their role in CNS disorders such as centrally mediated chronic pain (e.g., fibromyalgia), PTSD, and cognitive impairment.
